# Novel therapies in genitourinary cancer: an update

**DOI:** 10.1186/1756-8722-1-11

**Published:** 2008-08-11

**Authors:** David Chu, Shenhong Wu

**Affiliations:** 1 Department of Medicine, Stony Brook University Medical Center, Stony Brook, New York, USA

## Abstract

In recent years, new treatment for renal cell carcinoma (RCC) has been a spotlight in the field of cancer therapeutics. With several emerging agents branded as 'targeted therapy' now available, both medical oncologists and urologists are progressively more hopeful for better outcomes. The new remedies may provide patients with improved survival and at the same time less toxicity when compared to traditional cytotoxic agents. This article will center on current and emerging treatment strategies for advanced RCC and other GU malignancies with updates from 2008 annual ASCO meeting.

## Renal cell cancer

For many years the treatment of advanced RCC was limited to the immunotherapy with interleukin-2 (IL-2) and interferon-α (IFN-α). The advent of targeted agents beginning in late 2005 filled a prolonged void of relatively fruitless therapies. The approval of the tyrosine kinase inhibitor sorafenib in December, 2005 both opened the door for the entrance of numerous other agents that became readily available, and also gave new optimism to patients afflicted with RCC.

The incidence of RCC has nearly doubled in the past two decades; it comprises approximately 2% of all human malignancies [1]. Across the world, more than 100,000 people will die annually from RCC [2]. The 5-year survival for patients with RCC has increased 2-fold over the past fifty years to almost 62%; this is likely due to earlier detection of the tumor, which has led to a more prompt initiation of oncologic treatment [3]. However, the 5-year survival rate for patients with metastatic disease continues to remains dismal at <2% [4].

Traditionally, surgical resection of RCC was ultimately the only consistent curative option for patients with localized disease, but the efficacy of available treatments for widespread disease remained marginal at best. Historically, spontaneous remissions observed in patients with RCC were thought to be a product of an immune response; this became the foundation behind the development of immune therapy beginning in the early 1980's [5]. Cytokine therapy with IL-2 and IFN-α became widely accepted as the standard of care for patients with extensive disease. High dose bolus IL-2 can trigger an immune response against RCC, but it will achieve a response rates only between 10 to 20% in patients, and is associated with severe life-threatening toxicities [6]. Monotherapy with IFN-α heralded a more tolerable toxicity profile but also shared a limited response rate of less than 10% [7].

New biologic agents with promising anti-tumor properties and a more favorable toxicity profile stemmed from the study of patients with Von Hippel-Lindau (VHL) disease, a familial cancer syndrome [8]. A better understanding of the VHL tumor suppressor gene and its role in up-regulating growth factors associated with angiogenesis spawned a novel approach to treating RCC that was dramatically different from traditional therapies [9]. Growth factors such as vascular endothelial growth factor (VEGF), epidermal growth factor (EGF), and platelet-derived growth factor (PDGF) and their downstream signalling pathways including phosphatidylinositol 3-kinase (P13K) and mammalian target of rapamycin (mTOR) became new targets in the crusade against RCC. Here we have concisely reviewed recent progress in the targeted treatment of RCC and summarized its results in Table 1.

**Table 1:** Indication and efficacy for selected agents in advanced RCC

Indications	Agent	Target	Efficacy
**1^st ^line**			

Poor risk	Temsirolimus	mTOR	Survival benefit (HR: 0.73) [33]
Good risk and clear cell	High dose IL-2	Immunomodulation	ORR: 14% (potential durable response) [99,100]
Good and intermediate risk	Sunitinib	VEGFR 1,2,3, PDGFR α,β Abl, Src	Survival benefit (HR: 0.82) [22]
Good and intermediate risk	Bevacizumab/Interferon	VEGF/Immunomodulation	PFS benefit (HR: 0.63) [49]

**2^nd ^line**			

Prior cytokine therapy	Sorafenib	VEGFR 1,2,3, PDGFR, C-kit, Flt-3, RET	PFS benefit (HR: 0.44) [12]
Prior TKIs	Everolimus	mTOR	PFS benefit (HR: 0.30) [35]

**Clinical trials**			

	Thalidomide	Immunomodulation and angiogenesis	ORR: 0% [42]
	Lenalidomide	Immunomodulation and angiogenesis	ORR: 11% [44]
	Axitinib	VEGFR 1,2, PDGFR, C-kit	ORR: 21–44.2% [24,101]
	Pazopanib	VEGFR 1,2,3, PDGFR, C-kit	ORR: 34.7% [27]
	Cediranib	VEGFR 1,2,3, PDGFR, C-kit, FLT-4	ORR: 38% [29]
	G250	CA IX	ORR: 10% [57]
	Ixabepilone	Cytotoxic	ORR: 12.6% [58]

**Abbreviations**: RCC, renal cell cancer; mTOR, mammalian target of rapamycin; VEGFR, vascular endothelial growth factor receptor; PDGFR, platelet derived growth factor receptor; c-KIT, stem-cell growth factor receptor; Flt-3 and -4, Fms-like tyrosine kinases 3 and 4; HR, hazard ratio; ORR, objective response rate; PFS, progression-free survival.

## Tyrosine kinase inhibitors (TKI)

### Sorafenib

Sorafenib is a small molecule multi-kinase inhibitor with effects on tumor cell proliferation and tumor angiogenesis. It was initially developed as an inhibitor of Raf kinase, but also has broad spectrum activity against multiple tyrosine kinases including vascular endothelial growth factor receptor family (VEGFR 1, 2, 3), platelet-derived growth factor receptor family (PDGFR-β), stem-cell growth factor receptor (c-KIT), Fms-like tyrosine kinase 3 (Flt-3), and the receptor encoded by the ret proto-oncogene (RET) [10]. Its efficacy for RCC was first shown in a phase II randomized discontinuation trial [11], and became the first drug approved for the treatment of advanced RCC since the approval of interleukin-2 in 1992. The median progression-free survival (PFS) was significantly longer in patients treated with sorafenib (24 weeks) than placebo (6 weeks). Based on these results, a randomized phase III trial was performed comparing sorafenib with placebo in patients with cytokine-refractory metastatic clear cell RCC. It demonstrated superiority of sorafenib which carried a median PFS of 5.5 months versus 2.8 months when compared to placebo [12]. The final analysis from this study revealed a median survival of 17.8 months in patients receiving sorafenib versus 15.2 months in patients receiving placebo. Although this was not statistically significant, further analysis showed a confounding effect of crossover from placebo to sorafenib [13].

Further studies are underway defining the role of sorafenib in the first-line setting. In a phase II randomized trial in patients with previously untreated advanced RCC, sorafenib was compared to IFN-α. There was no significant difference in PFS between sorafenib and IFN-α. A PFS benefit was observed in patients who crossed to sorafenib after progression on IFN-α. Patients who were dose escalated to 600 mg bid after disease progression had disease stabilization for a further 3.6 months [14]. These studies reinforced the rationale behind continuing large multi-center trials utilizing sorafenib in advanced RCC.

The role of sorafenib in RCC patients refractory to other anti-VGF therapy is not clear. In a multicenter prospective trial of sorafenib in patients with metastatic RCC refractory to prior sunitinib or bevacizumab therapy presented at ASCO 2008, administration of sorafenib proved to be feasible with a reduction of tumor burden observed [15].

Sorafenib-associated adverse effects, which are different from that of classical chemotherapy, include predominantly gastrointestinal and cutaneous manifestations with hand-foot skin reaction (HFSR) and diarrhea being the most common events. A recent meta-analysis of 11 randomized trials showed that among 4883 patients receiving sorafenib, the summary incidences of all-grade and high-grade HFSR were 33.8% and 8.9% respectively. Sorafenib was associated with a significantly increased risk of all-grade HFSR when compared with controls (RR: 6.6, 95% CI: 3.7 to 11.7, p < 0.001). Interestingly, the incidence of HFSR is significantly higher in patients with RCC than non-RCC malignancies (RR: 1.52, 95% CI: 1.32–1.75%, p < 0.001) [16].

### Sunitinib

Similar to sorafenib, sunitinib is a small molecule inhibitor of multiple tyrosine kinases including VEGFR, PDGFR α and β, Src, Abl, insulin-like growth factor receptor-1, and fibroblast growth factor receptor-1 tyrosine kinase [17]. A phase I study revealed initial activity in RCC and gastrointestinal stromal tumors [18]. Two phase II studies established its potent activity in RCC. The initial study enrolled patients predominantly pretreated with immunotherapy, and showed a 40% objective response rate and a median time to progression of 8.7 months [19]. The following study shared similar results with a response rate of 34% and median progression-free survival of 8.3 months [20]. Toxicities reported from these trials were predominantly fatigue, nausea, diarrhea and stomatitis. A handful of patients was found to have a significant decrease in myocardial ejection fraction leading termination of drug administration [20].

The success of sunitinib in the treatment of cytokine-refractory RCC led the way for the phase III study in the first-line setting [21]. Seven hundred fifty previously untreated patients with RCC were randomized to sunitinib or IFN-α, and the survival results were recently updated at ASCO 2008 [22]. The objective response rate (ORR) was significantly increased with sunitinib (47%) when compared to IFN-α (12%). The median PFS was also significantly higher in the sunitinib group (11 months) when compared to the IFN-α group (5 months). Median overall survival (OS) was also greater in the sunitinib group (26.4 months) compared with the IFN-α group (21.8 months) with a hazard ration of 0.82 [22]. Quality of life was significantly better in the sunitinib group. This trial ultimately established sunitinib as the first-line therapy for advanced RCC.

Sunitinib is also being analyzed in the adjuvant setting. The 'Sunitinib Treatment of Renal Adjuvant Cancer'(S-TRAC) study is a randomized double blind placebo controlled trial examining RCC patients with locally advanced but not metastatic disease 10 weeks following radical nephrectomy. Patients will be treated with sunitinib or placebo for 1 year, and disease-free survival and OS will be among the endpoints evaluated [23]. Sunitinib is also being evaluated in combination with other agents in the treatment of advanced RCC.

### Axitinib

Axitinib (AG013736) is an orally active multi-kinase inhibitor that inhibits the receptor tyrosine kinases VEGFR 1 and 2, PDGFR and c-KIT. In a phase II trial of 52 patients with advanced RCC, an ORR of 44.2% was observed with a similar safety profile as other tyrosine kinase inhibitors [24]. Another phase II study of 62 patients refractory to tyrosine kinase inhibitors showed promising results with 21% of patients achieving partial response and 34% achieving stable disease [25]. Results of a phase II multicenter trial using axitinib in patients with metastatic RCC refractory to cytokines and sorafenib, sorafenib and sunitinib, or sorafenib alone was presented at ASCO 2008 [26]. In patients refractory to sunitinib and sorafenib, the ORR was 7% and median PFS was 7.1 months; in patients refractory to cytokines and sorafenib, the ORR was 28% and median PFS was 9 months; in patients refractory to sorafenib alone, the ORR was 27% and the median PFS was 7.7 months. It was concluded that axitinib has anti-tumor activity in patients refractory to other tyrosine kinase inhibitors supporting the notion that there is an absence of total cross-resistance between axitinib and other TKIs.

### Pazopanib

Pazopanib (GW786034) is a selective multi-kinase inhibitor of VEGFR 1–3, PDGFR, and c-kit. Results of a phase II randomized discontinuation trial in 225 patients with metastatic RCC in the first and second line setting revealed a partial response in 61 patients (27%) and stable disease in 104 patients (46%). Common adverse events included diarrhea, cutaneous manifestations, and hypertension [27]. The results were recently updated at ASCO 2008, and illustrated an ORR of 34.7% (CR: 1.3%, PR: 33.3%). Stable disease was achieved in 44.5% of patients, and median PFS was 11.3 months [28].

### Cediranib

Cediranib (AZD2171) is an orally available selective inhibitor of VEGFR 1, 2, 3, PDGFR, c-kit, and FLT-4. At ASCO 2008, a phase II trial was presented that included 43 patients with advanced untreated RCC. Partial responses were observed in 12 patients (38%) and stable disease was observed in 15 patients (47%). Overall tumor control rate was observed in 27 patients (84%), and median PFS was 8.7 months. Treatment-related adverse events were tolerable, and included hypertension and fatigue [29].

## Mammalian target of rapamycin inhibitors

### Temsirolimus

Temsirolimus is a highly specific inhibitor of the mammalian target of rapamycin (mTOR), a large polypeptide kinase which forms part of the PI3K/Akt pathway. It is a central regulator of intracellular signaling pathways involved in tumor cell growth, proliferation and angiogenesis [30,31]. Its effect on advanced refractory RCC patients was demonstrated initially in a randomized phase II trial of patients with metastatic RCC. Although the ORR was only 7%, the main benefit was disease stabilization with 51% of patients achieving a response or stable disease for more than 6 months [32]. This trial led the way to its evaluation in a phase III clinical trial in patients with previously untreated intermediate or poor-risk advanced RCC [33]. The study compared the use of single agent temsirolimus, single agent IFN-α, and a combination of temsirolimus plus IFN-α in these patients. Temsirolimus monotherapy significantly prolonged the median OS compared to IFN-α as a single agent (10.9 months vs 7.3 months). Furthermore, patients receiving temsirolimus had a significantly longer PFS than patients receiving IFN-α (3.7 months vs 1.9 months). The combination group also shared a PFS of 3.7 months. It was approved for the treatment of advanced RCC in May of 2007. Toxicities from single-agent temsirolimus included fatigue (54%), nausea (37%), rash (37%) and dyspnea (30%). Temsirolimus showed a survival benefit in this group of intermediate to poor risk patients with advanced RCC, and future studies in good risk populations would be of interest.

### Everolimus

Everolimus (RAD001) is an oral mTOR inhibitor that has gained attention in recent months. It was tested in the first- and second-line setting in 37 advanced RCC patients in a phase II clinical trial [34]. Twelve of these patients had partial responses, and 19 of them were stable for more than 3 months. A phase III randomized double-blind placebo controlled trial presented at ASCO 2008 showed significant PFS over placebo in patients with prior treatment of tyrosine kinase inhibitors including sorafenib and sunitinib [35]. Two hundred seventy two patients were randomized to everolimus and 138 patients to placebo; results showed a significant difference in PFS in patients receiving everolimus as compared to placebo (4 months vs 1.9 months). Its safety profile was favorable with the most common adverse events being stomatitis, anemia and asthenia. This study has established the role of everolimus as second or third line therapy after treatment failure of tyrosine kinase inhibitors.

Everolimus was also evaluated in combination therapy with sorafenib in a phase I study as well as with bevacizumab in a phase II study at ASCO 2008 with both trials showing anti-tumor activity and tolerability of combination therapy [36,37].

## Immunomodulators

### Thalidomide and its analogs

Thalidomide is a potent immunomodulatory drug with antiangiogenic properties. Initial studies showed thalidomide to have potential activity in advanced RCC [38-41]. However, a randomized phase II study of 60 patients refractory to immunotherapy did not show promising results [42]. These patients were randomized to receive either thalidomide or medroxyprogesterone. All patients receiving medroxyprogesterone experienced progression of disease, while only 3 patient in the thalidomide arm achieved stable disease at 3 months [42]. Further studies using thalidomide in combination with other agents are ongoing [43].

Lenalidomide is a thalidomide derivative with potent immunomodulatory and antiangiogenic properties. In a recent open-label single center phase II trial, 40 newly diagnosed RCC patients were treated with oral lenalidomide monotherapy [44]. A complete response was seen in one patient (3%), partial response was seen in 3 patients (8%), and stable disease was observed in 21 patients (53%). The most common adverse events were fatigue, neutropenia and thrombocytopenia. The activity in RCC has also been observed in other small phase II trials [45,46]. Further studies will be needed to assess its efficacy in advanced RCC.

## Monoclonal antibodies

### Bevacizumab

Bevacizumab is a humanized monoclonal antibody that inhibits tumor angiogenesis by targeting VEGF. It neutralizes all of the major isoforms of VEGF [47]. Initial evaluation began with a randomized, double-blind phase II trial comparing bevacizumab at low-dose and high-dose to placebo in 116 patients with cytokine-refractory RCC. Bevacizumab treatment led to significant prolonged time to progression of disease in the high-dose group compared to placebo (4.8 months vs 2.5 months) [48]. These results were the first to prove the concept that the VEGF signaling pathway is important for the progression of RCC in humans. It also led to evaluation of bevacizumab in combination with other therapeutic agents. The AVOREN trial was a randomized, double-blind phase III trial (n = 649) investigating the standard therapy of IFN-α plus placebo versus IFN-α plus high dose bevacizumab [49]. The median PFS for patients was significantly prolonged in the patients in the bevacizumab arm when compared with IFN-α alone (10.2 months vs 5.4 months). In addition, the ORR was also greater in the bevacizumab arm when compared with IFN-α alone (31% vs 13%). Serious adverse events were more common in patients treated with bevacizumab plus IFN-α when compared with IFN-α alone (29% vs 16%).

Bevacizumab therapy was also evaluated in pre-surgical patients with metastatic RCC at ASCO 2008 [50]. The study was initiated to evaluate the safety and efficacy of bevacizumab therapy prior to cytoreductive nephrectomy. Of the 48 patients that were enrolled in the study, 1 patient achieved a complete response of the target lesion, 4 achieved a partial response, and 27 achieved stable disease. The ORR was 10.9%. It was concluded that bevacizumab treatment prior to nephrectomy was feasible and a safe treatment option. Bevacizumab has also been evaluated in combination with other agents including IL-2, sorafenib, sunitinib, temsirolimus, and erlotinib [51-55].

### G250

G250 is a chimeric monoclonal antibody directed against carbonic anhydrase IX, a heat-sensitive surface antigen which is ubiquitously expressed in RCC [56]. In an early clinical trial accruing 35 patients with metastatic RCC, daily low dose IL-2 was combined with G250 and the result was optimistic. Three patients had partial responses and 5 patients had stable disease for at least 6 months. The median OS was 22 months [57]. Phase III randomized trials are currently underway further evaluating G250 in RCC.

## Epothilones

Epothilones are a new class of cytotoxic agents derived from the fermentation of broth of the myxobacterium *Sorangium cellulosum*. Ixabepilone is a semisynthetic analog of epothilone B analog, a non-taxane microtubule-stabilizing agent active against cancers insensitive to paclitaxel. On October 16, 2007 it was approved for the treatment of metastatic, refractory breast cancers in combination with capecitabine. A phase II study was presented at ASCO 2008 investigating its activity in metastatic RCC [58]. Eighty seven patients with advanced RCC received ixabepilone, and results revealed an ORR of 12.6% with 1 patient achieving a complete response and 10 patients achieving partial responses. Median response duration was 5.5 months, and median OS of patients with clear cell histology was 19.25 months. Treatment related adverse events were predominantly grade I and II. It is the first time that a cytotoxic chemotherapeutic agent showed significant efficacy in RCC. This agent may be combined with other novel agents in the future.

### Summary

With multiple therapeutic options available for advanced RCC, the optimal treatment is unclear. Based on current evidence, the following approaches may be followed, as summarized in Table 1. In the first line setting, high-dose IL-2 may be used for patients with good prognostic features and clear cell histology; it is the only therapy which may provide a benefit of long-term complete response. Temsirolimus should be the standard therapy for patients with poor prognostic features because of survival advantage. Sunitinib should be the drug of choice in patients with good-intermediate prognostic features, as the updated data from 2008 ASCO has shown that it improves survival. Bevacizumab in combination with interferon α has been shown to increase progression-free survival, and may be considered in patients who do not tolerate sunitinib. In the second line setting, sorafenib and everolimus may be used in patients with prior cytokine therapy and prior TKI treatment respectively due to progression-free survival benefit. The use of other agents should be in the setting of clinical trials.

## Prostate cancer

Prostate cancer is the most common non-cutaneous malignancy in men. For advanced disease, eventually almost all of patients will develop resistance to androgen deprivation therapy based on medical or surgical castration. Historically, patients with castration-refractory prostate cancer (CRPC) had a median survival of approximately 12 months with scant treatment options [59]. Chemotherapy was previously considered to have an insignificant role in CRPC; however in 2004, two pivotal phase III trials (SWOG 9916 and TAX 327) led to the approval of docetaxel in combination with prednisone as the standard treatment of CRPC, and opened the door to a new era of prostate cancer treatment [60,61]. Since the approval of docetaxel, treatment approaches to CRPC centered around two tactics: the first was to utilize docetaxel as a starting place for combination therapy. The second was to take advantage of novel therapeutic agents. Here we will mainly focus on novel agents with activity for CRPC. Below we have reviewed recent clinical trials on the use of novel agents in the treatment of CRPC (efficacy data summarized in Table 2).

**Table 2:** Efficacy of novel agents in CRPC.

Mechanism	Agent	PSA Response Rate
**Cytotxic**	Satraplatin	33% 1^st ^line [62] 25% 2^nd ^line [64]
	Patupilone	42% 2^nd ^line [80]
	Ixabepilone	33% 1^st ^line [76] 17–20% 2^nd ^line [77]
	Sagopilone	29% 1^st ^line [81]
**Immunomodulation**	Thalidomide	18% 2^nd ^line [68]
**Androgen synthesis inhibition**	Abiraterone Acetate	60% 1^st ^line [83] 40% 2^nd ^line [84]
**Combinations**		
Cytotoxic/Immunomodulation	Docetaxel/Thalidomide	53% 1^st ^line [69]
Cytotoxic/VEGFR/Immunomodulation	Bevacizumab/Thalidomide/Docetaxel	88% 1^st ^line [70]
Cytotoxic/VEGFR	Bevacizumab/Docetaxel/Estramustane	81% 1^st ^line [67]

**Abbreviations**: CRPC, castration-refractory prostate cancer; VEGFR, vascular endothelial growth factor receptor.

### Satraplatin

Satraplatin is an oral third generation platinum compound with activity against a variety of solid tumors. It has preclinical antitumor activity comparable with that of cisplatin but a better toxicity profile. In a randomized phase III trial led by European Organization for Cancer Research (EORTC) with a target sample size of 380 patients of CRPC, the trial was closed to further accrual by the sponsoring company after 50 randomized patients. An ad hoc analysis demonstrated a significant median PFS advantage of satraplatin over prednisone itself (5.2 months vs 2.5 months) as well as a significant improvement in PSA response (33% vs 9%) [62].

This data led to the evaluation of satraplatin plus prednisone in a multi-national placebo-controlled phase III trial known as the SPARC (Satraplatin and Prednisone Against Refractory Cancer) which pitted satraplatin plus prednisone against placebo plus prednisone as second-line therapy in patients with CRPC [63]. Initial results appeared promising with a PFS in favor of satraplatin (11.1 weeks vs 9.7 weeks), but updated results at ASCO 2008 were disappointing. Despite the fact that there was significant improvement in the satraplatin group in PFS, time to progression, PSA response, objective tumor response, pain response, and duration of pain response, there was no improvement in OS (61.3 weeks vs 61.4 weeks) [64]. Nevertheless, favorable trends were observed in the subgroup of patients that had received prior docetaxel therapy. The most common toxicities included myelosuppression and thrombocytopenia. It was concluded that satraplatin was a tolerable treatment which improved PFS, but studies are underway attempting to pinpoint a subgroup of patients who may benefit from this treatment.

### Angiogenesis Inhibitors

Angiogenesis is an important and tightly regulated factor in the development of tumor growth and metastasis [65]. Several angiogenesis inhibitors have been undergoing evaluation in CRPC, including bevacizumab, thalidomide, sorafenib, sunitinib, and cediranib (AZD2171).

The efficacy of bevacizumab as a single agent in CRPC is marginal [66]. However, significant activity was observed when it was combined with docetaxel and estramustine in the Cancer and Leukemia Group B (CALBG) 9006 trial [67]. The results showed a 50% or greater PSA decline in 81% of 79 chemotherapy naïve patients with a median time to progression of 9.7 months and median OS of 21 months. This led to the initiation of the ongoing CALGB 90401 phase III trial treating CRPC patients with docetaxel and prednisone with or without bevacizumab.

Thalidomide has also been of interest in CRPC because of its anti-angiogenic and immunomodulatory properties. In a randomized phase II trial, 63 patients with CRPC were treated with either a low-dose or a high-dose of thalidomide [68]. Of 50 patients in the low dose arm, 9 (18%) had a ≥50% decrease in PSA which was maintained at least 28 days. There were 4 patients who maintained this decrease in PSA for at least 150 days. These results led the investigators to perform a randomized phase II trial including 75 chemotherapy naïve CRPC patients treated with either docetaxel or docetaxel plus thalidomide [69]. Patients were randomized in a 1:2 fashion with 25 patients receiving docetaxel alone and 50 patients receiving the combination. Of 24 patients receiving docetaxel alone, 37% had a PSA decrease >50% while of 47 patients in the combination arm 53% had a PSA decrease of > 50%. The 18-month survival was 68% for the 2 groups, with a median progression-free survival of 3.7 months in the single agent arm versus 5.9 months in the combination arm; OS was 14.7 months versus 28.9 months. Although the combination was tolerated well, increased thromboembolic events were observed in the combination arm.

These studies involving angiogenesis inhibitors prompted evaluation of both agents together in combination with docetaxel. At ASCO 2008, a single arm phase II trial using thalidomide, docetaxel and bevacizumab was presented [70]. Sixty patients with chemotherapy naïve CRPC were treated with the combination of docetaxel, thalidomide and bevacizumab with the addition of enoxaparin for thrombosis prevention as well as pegfiligrastim support for neutropenia. 88% of patients had a PSA decline of >50%, and 71% of patients had a PSA decline of >80%. The ORR was 63%, and the median PFS was estimated to be 18.2 months. Toxicities included febrile neutropenia, osteonecrosis of jaw, syncope, GI perforation and thrombosis. These results showed a promising durable control of the disease with the regimen, and future studies may need to explore a combination of angiogenesis inhibitors with a better toxicity profile.

Several TKIs including sorafenib, sunitinib, and cediranib have also been evaluated in phase II studies showing promising activity in CRPC with and without prior docetaxel treatment [71-75]. Interestingly, PSA does not appear to be a good predictor for disease progression in patients treated with sorafenib, suggesting that clinical and radiographic evidence may be a better marker to assess the activity of these new agents.

### Epothilones

With docetaxel's success in CRPC, the use of epothilones became attractive as this class of agents carries a mechanism of action similar to the taxanes. Of the epothilones, ixabepilone gained attention in the treatment of CRPC after a phase II SWOG trial of 42 chemotherapy naïve CRPC patients who received ixabepilone in the first line setting. Fourteen of the evaluable patients (33%) had a confirmed PSA response. The estimated PFS was 6 months, and the median survival was 18 months [76]. The most common toxicities were neuropathy and myelosuppression. This led to a multi-center, non-comparative, double crossover phase II study which evaluated the safety and activity of ixabepilone in patients with CRPC refractory to docetaxel-based therapy [77]. Patients were randomized to either ixabepilone alone or mitoxantrone plus prednisone, and patients who progressed while on treatment or discontinued treatment for toxicity were allowed to crossover to the other study arm. The median survival was similar in the ixabepilone and mitoxantrone arms (13 months vs 12.5 months). The PSA response for the ixabepilone group was 17% while the PSA response in the mitoxantrone group was 20%. These findings showed that these two agents had only marginal activity in the docetaxel-refractory CRPC setting. At ASCO 2008, a combination of these two agents was evaluated in a phase I study of ixabepilone, mitoxantrone and prednisone in patients with metastatic CRPC refractory to docetaxel-based therapy [78]. Thirty two patients were treated at 6 distinct dosing levels. Eight patients developed a PSA response, however 20 patients suffered high grade neutropenia. The results suggest that the combination of ixabepilone with mitoxantrone plus prednisone is feasible but may require pegfilgrastim support in the future.

A second study presented at ASCO 2008 updated results of E3803, a phase II study of ixabepilone administered on a weekly dosing schedule as opposed to every 3 weeks [79]. Patients with metastatic CRPC (n = 96) that were chemotherapy-naïve or pretreated with taxane-based therapy were treated with ixabeppilone on a weekly basis. PSA response was achieved in 32% of chemotherapy-naïve and 22% of pretreated patients. This study showed that ixabepilone administered on a weekly basis was feasible.

Patupilone is another epothilone with broad spectrum pre-clinical activity in taxane-resistant models. In a phase II study in patients with CRPC refractory to docetaxel presented at ASCO 2008, 33 patients received patupilone every 3 weeks and at the time of the presentation 63% of patients achieved a PSA decline of >30% while 42% of patients achieved a PSA decline of >50%. A confirmed PSA response was seen in 26% of patients [80]. These results are encouraging as the study continues to accrue.

Sagopilone (ZK-EPO) is a fully synthetic epothilone B analogue. It was evaluated in a phase II study (n = 29) in chemotherapy-naïve CRPC patients in combination with prednisone. It showed that 21% of patients had a PSA response of >50%, and 58% of patients had a PSA response of >30% [81]. High-grade toxicities included neuropathy, fatigue, diarrhea and dizziness.

### Cilengitide

Cilengitide (EMD 1219749) is a potent selective αVβ3 and αVβ5 integrin antagonist. Integrins are cell surface receptors that mediate a variety of cell activities including endothelial cell proliferation and migration. αVβ3 is important in bone metabolism, and may play a role in CRPC growth in bone. At ASCO 2008, a phase II randomized trial was presented in which 44 asymptomatic chemotherapy-naïve patients with metastatic CRPC were randomized to high-dose and low-dose cilengitide [82]. The primary endpoint was 6-month objective progression rate excluding PSA. There were stable disease in 27% of patients in the low dose arm and 36% of patients in the high dose arm. Cilengitide was well tolerated and had a favorable safety profile. The improvement in objective progression rate with the high-dose arm was marginal and therefore was not pursued any further. Neither dose succeeded in decreasing the 6 month objective progression rate.

### Abiraterone acetate

One of the most exciting findings in the field of prostate cancer is the recognition that persistent androgen signaling remains critical in CRPC. Approaches to targeting androgen signaling including androgen synthesis and receptor blockage have drawn renewed interest. Abiraterone acetate (AA), a selective inhibitor of 17-α hydroxylase and C17, 20-Lyase which is a dual enzyme responsible for adrenal androgen synthesis, has shown a promising therapeutic significance to intervene this pathway. The mechanism of action for abiraterone may be similar to ketoconazole, which inhibits multiple adrenal CYP enzymes including CYP17, and is used as second line hormonal treatment for CRPC. At 2008 ASCO, a study of AA was presented in patients who failed androgen deprivation therapy [83]. These patients were enrolled in two parallel trials; the first was a phase I/II study in chemotherapy-naïve CRPC patients and the second was a phase II study in CRPC patients refractory to taxane therapy. A PSA response of >50% was seen in 60% of the patients in the chemotherapy-naïve arm and in 40% of the pretreated arm. The median time to PSA progression was 252 days in the chemotherapy-naïve arm and 167 days in the pretreated arm. An ultrasensitive serum testosterone assay was used to confirm significant testosterone suppression beyond conventional androgen deprivation therapy. Toxicities stemmed from mineral corticoid excess such as hypertension, hypokalemia and fluid retention but were tolerable. A second study was a combination of AA with prednisone in patients with CRPC after failure of docetaxel based chemotherapy [84]. Forty three patients were given AA with prednisone, and at 3 months 14 of 35 evaluable patients (40%) achieved a decline in PSA > 50%. A third study presented at ASCO 2008 confirmed its activity in patients with ketoconazole-refractory disease, suggesting no cross-resistance between the two agents [85].

## Bladder cancer

Despite advances in the treatment of superficial bladder cancer, many patients eventually perish from metastatic disease. It is the fourth most common cancer afflicting men and the ninth most common cancer afflicting women [1]. First-line therapy for metastatic bladder cancer has been cisplatin-based therapy for the past 20 years. In recent years, the combination of methotrexate, vinblastine, doxorubicin and cisplatin (M-VAC) has been the standard of care for the treatment of metastatic bladder cancer, but the regimen's unfavorable toxicity has prompted a search for an alternative treatment. A recent large randomized phase III trial comparing M-VAC with the combination of cisplatin and gemcitabine (CG) offered a viable alternative [86]. The study showed no statistical difference in OS or ORR, but a more favorable toxicity profile for the CG group thus replacing M-VAC as the standard of care for patients with advanced bladder cancer. Despite these findings, treatment with these regimens rarely provides a prolonged relapse free or overall survival which has stimulated a search for more efficacious treatment including cytotoxic and targeted agents.

### Pemetrexed

Pemetrexed is an anti-folate anti-metabolite with multiple enzyme targets involved in both pyrimidine and purine synthesis. Its efficacy in bladder cancer has been evaluated in a phase II study in the second-line setting for the treatment of advanced bladder cancer [87]. The study demonstrated an ORR of 28% and an OS of 9.6 months. In addition, it has also been evaluated in combination with gemcitabine in a phase II study of 63 patients with advanced bladder cancer in the first line setting [88]. The ORR of this combination was 26.5%, but toxicities were significant. The results were disappointing in that the ORR and OS appear inferior to standard cisplatin-based regimens.

### Vinflunine

Vinflunine is a novel vinca alkaloid that inhibits tubulin and acts to inhibit assembly of microtubules. It has recently gained attention in the treatment of advanced bladder cancer after phase I studies confirmed anti-tumor activity. In a phase II study treating 51 patients who had failed first-line cisplatin containing regimens, vinflunine resulted in an ORR of 18%, a PFS of 3 months and a median OS of 6.6 months [89]. This study led to the initiation of a phase III trial of vinflunine plus best supportive care (BSC) versus BSC alone as second-line therapy after failure of a cisplatin-containing regimen in transitional cell carcinoma of the urothelium (TCCU) presented at ASCO 2008 [90]. Three hundred seventy patients were randomized in a 2:1 fashion to vinflunine plus BSC vs BSC, and the results showed that patients included in the vinflunine group achieved a median 2 month overall survival advantage (6.9 months vs 4.6 months) but was not statistically significant (p = 0.29). However, the planned multivariate analysis adjusting for prognostic factors showed improved survival with vinflunine (HR = 0.77, p = 0.036). These results may support the role of vinflunine as a standard second line treatment for advanced TCCU.

### Traztuzumab

Trastuzumab is a humanized monoclonal antibody against Her-2/neu approved for the treatment of Her-2/neu positive breast cancer patients. In a phase II study evaluating trastuzumab's activity in advanced Her-2/neu positive bladder cancer, 44 patients with Her-2/neu positive bladder cancer were treated with a combination of paclitaxel, carboplatin, gemcitabine and trastuzumab [91]. The results demonstrated an ORR of 70% with a median time to progression of 9.3 months and an OS of 14.1 months, with 23% of patients experienced cardiotoxicity. Lapatinib, an inhibitor of HER-2/neu tyrosine kinase,, has also been evaluated in a phase II trial, and it showed ORR in 14% of patients and a median time to progression equivalent to other second line therapies (8.6 weeks) [92]. These results warrant further study of targeting Her-2 signalling pathway.

### TKI

The efficacy of TKIs in advanced bladder cancer has been explored by some studies presented at ASCO 2008. Sunitinib's activity in relapsed or refractory bladder cancer was evaluated by a phase II study [93]. In this study, 45 patients who received <4 previous chemotherapy regimens were treated with sunitinib as a single-agent. Results showed that 3 patients achieved a partial response and 11 patients achieved stable disease. Radiographic regression was observed in liver, lung, bone, bladder, soft tissue and lymph node lesions. This trial demonstrated that sunitinib does have activity in bladder cancer. Sorafenib was also evaluated at in a phase II trial (E1804) where 27 patients were accrued for treatment [94]. The first stage of accrual was suspended for a pre-planned efficacy evaluation where criteria for continuing on to the second stage of accrual were not met. For evaluable patients, no objective responses were observed and median OS was 6.8 months. It seems that sorafenib as a single agent had minimal activity in bladder cancer.

## Testicular cancer

Testicular cancer is the most common cancer diagnosis in males between the age of 15 and 35 years [1]. The majority of testicular tumors are germ cell tumors (GCT). The cure rate is the highest of any solid organ tumor and stems from the utilization of highly effective chemotherapy.

The treatment for early stage GCT has been controversial for both seminoma and non-seminomatous tumors. Treatment options after resection of a stage I seminoma include active surveillance, radiation to the paraaortic lymph nodes, or single agent carboplatin. At the recent ASCO 2008, an updated analysis of the MRC/EORTC randomized trial (ISRCTN27163214) compared one course of carboplatin at AUC 7 with adjuvant radiation for stage I seminoma [95]. As relapses may occur 10 years following treatment, patients were continued to be followed for data collection. Patients (n = 1,447) were randomized in a 3:5 ratio (carboplatin:radiation). The relapse free rate (RFR) at five years was 95% for the carboplatin group and 96% for the radiation group. Only one death from seminoma was reported. However, there was an increased risk of developing a second GCT in the radiation arm as compared to the carboplatin arm (2 patients versus 15 patients). It was concluded that a single dose of carboplatin was not inferior to radiation therapy in stage I seminoma, and carboplatin therapy was associated with a significantly decreased risk of developing a second GCT.

For advanced GCT, the interest has been to find the best salvage treatment. There are mainly two approaches used in the second line setting; combination chemotherapy based on ifosfamide and cisplatin or high-dose chemotherapy (HDCT) with stem cell rescue. HDCT has been used successfully in GCT since the early 1980's, but its use has traditionally been limited by treatment-related toxicities and mortality. There is limited data comparing conventional chemotherapy and HDCT in the salvage setting. A retrospective match-pair analysis found HDCT to be more beneficial [96]. From two large databases, 193 patients with relapsed or refractory non-seminomatous GCT were identified. In 74 of those patients, salvage treatment by HDCT was to be administered. Patients were matched based on primary tumor location, response to first-line treatment, duration of this response and serum levels of the tumor. The analysis suggested a benefit from HDCT, with an estimated absolute improvement in event-free survival of between 6 and 12% and in overall survival of between 9 and 11% at 2 years. However, this conclusion is not supported by a phase III study. The trial (n = 280) was performed to compare conventional salvage chemotherapy to HDCT [97]. Patients were randomly assigned to receive either four cycles of cisplatin, ifosfamide and etoposide (or vinblastine) or three such cycles followed by high-dose carboplatin, etoposide and cyclophosphamide with stem cell support. Complete and partial response rates were similar in both treatment arms (56%). There was 3% treatment related deaths in the conventional arm and 7% treatment related deaths in the HDCT. There was no significant difference in 3 year event free survival or OS.

The German Testicular Cancer Study Group attempted a prospective study in the salvage setting comparing one cycle of conventional chemotherapy consisting of etoposide, ifosfamide and cisplatin (VIP) followed by one cycle of HDCT versus 3 cycles of VIP followed by once cycle of HDCT [98]. The study was stopped prematurely as a result of excess mortality deaths in the second arm, but the investigators found no difference in the survival probabilities between the two groups. With limited data comparing conventional chemotherapy with HDCT, this topic will remain controversial, and further studies will be needed to identify a selected group of patients who may benefit from HDCT.

## Conclusion

The treatment of advanced renal cell cancers has evolved dramatically with the use of new targeted agents including bevacizumab, sunitinib, sorafenib, temsirolimus, and everolimus. The challenge will be how to sequence or combine these new agents for optimal results. Better understanding of prostate biology has led to the development of new hormonal drugs and a variety of cytotoxic and targeted agents. With additional novel agents and combinations under evaluation, the future of GU oncology appears promising and exciting.

## List of abbreviations used

ASCO: American society of clinical oncology; CALBG: Cancer and Leukemia Group B; EORTC: European Organization for Cancer Research; RCC: renal cell carcinoma; CRPC: Castration-refractory prostate cancer; GCT: Germ cell tumors; IL-2: Interleukin-2; IFN-α: Interferon-α; HDCT: High-dose chemotherapy; VIP: etoposide, ifosfamide and cisplatin; VHL: Von-Hippel-Lindau; VEGF: Vascular endothelial growth factor; EGFR: Epidermal growth factor receptor; PDGF: Platelet-derived growth factor; PI3K: Phosphatidylinositol 3-kinase; mTOR: mammalian target of rapamycin; TKI: Tyrosine Kinase Inhibitors; VEGFR: Vascular endothelial growth factor receptor; PDGFR: Platelet-derived growth factor receptor family; c-KIT stem-cell growth factor receptor; Flt-3: Fms-like tyrosine kinase 3; RET: the receptor encoded by the ret proto-oncogene; PFS: Progression-free survival; OS: Overall survival; ORR: Objective response rate; BSC: Best supportive care; RR: Relative risk.

## Competing interests

SW is a speaker for Pfizer Inc., and has received honoraria from Onyx Pharmaceuticals.

## Authors' contributions

DC and SW participated in the conception, drafting, and revision for the study. All authors read and approved the final manuscript.
